# 6-Shogaol Mitigates Sepsis-Associated Hepatic Injury through Transcriptional Regulation

**DOI:** 10.3390/nu13103427

**Published:** 2021-09-28

**Authors:** Xiaoxuan Guo, Jing Qiu, Yongzhong Qian

**Affiliations:** Institute of Quality Standard and Testing Technology for Agro-Products, Chinese Academy of Agricultural Sciences, Beijing 100081, China; guoxiaoxuan@caas.cn (X.G.); qiujing@caas.cn (J.Q.)

**Keywords:** 6-shogaol, LPS, liver injury, RNA sequencing, MAPK pathway, NFκB pathway

## Abstract

Sepsis-associated liver dysfunction presents a significant public health problem. 6-Shogaol is the key bioactive component in dry ginger, which has antioxidant and anti-inflammation capacity. The present study aims to investigate the preventive effect of 6-shogaol on sepsis-induced liver injury. 6-Shogaol was administered to mice for 7 consecutive days before being intraperitoneally injected with lipopolysaccharide (LPS). After 24 h, mice were sacrificed, and biochemical and transcriptomic analyses were performed. Our results demonstrated that 6-shogaol prevented LPS-induced impairment in antioxidant enzymes and elevation in malondialdehyde level in the liver. The hepatic inflammatory response was significantly suppressed by 6-shogaol through suppressing the MAPK/NFκB pathway. RNA-sequencing data analysis revealed that 41 overlapped genes between the LPS vs. control group and 6-shogaol vs. LPS group were identified, among which 36 genes were upregulated, and 5 genes were downregulated for the LPS vs. control group. These overlapped genes are enriched in inflammation-related pathways, e.g., TNF and NFκB. The mRNA expression of the overlapped genes was also verified in the LPS-induced BRL-3A cell model. In summary, 6-shogaol shows great potential as a natural chemopreventive agent to treat sepsis-associated hepatic disorders.

## 1. Introduction

Sepsis is a systematic inflammatory response caused mainly by bacteria. It is one of the most life-threatening illnesses among intensive care patients worldwide [1]. Clinically, the progression of sepsis starts from early sepsis, severe sepsis, septic shock, to multi-organ failure [1,2]. Lipopolysaccharide (LPS) is the major component of endotoxin in Gram-negative bacteria. LPS-injected mice model is a very common acute sepsis model [3,4]. The liver is one of the main target organs affected in this model since it plays a crucial role in immunological homeostasis and metabolism [5]. Liver damage and dysfunction in sepsis an independent risk factor for sepsis-induced death [6,7]. It has been demonstrated that patients with sepsis and early liver dysfunction exhibited significantly elevated mortality and poor prognosis [8]. Since sepsis is characterized by overwhelming immune response, combating the inflammation might reduce liver dysfunction and lower the mortality of sepsis [9].

A growing number of studies have indicated that phytochemicals from diets can help manage inflammatory processes; thus, the food industry has increasingly focused on developing functional food from natural sources to prevent inflammation and reduce risks for related diseases. Ginger (*Zingiber officinale*) is consumed worldwide as a flavoring spice and as a traditional medicinal herb. Ginger is reported to possess antioxidative, anti-inflammatory, and antitumor properties [10,11,12]. 6-Shogaol is a major bioactive component derived from dry ginger. A number of studies have demonstrated the strong anti-inflammatory property of 6-shogaol [13,14,15,16]. For instance, 6-shogaol can inhibit the production of proinflammatory cytokines through regulation of NFκB and phosphorylation of JNK in RAW 264.7 and HMC-1 cells [14,17]. 6-Shogaol attenuates neuroinflammation and cognitive deficits in animal models of dementia [15]. It has also been reported as the most potent anti-inflammatory and anti-oxidant component in ginger [18]. However, the protective role of 6-shogaol on sepsis-associated liver injury has been poorly elucidated. Therefore, we applied the LPS-induced mice sepsis model and firstly detected hepatic whole-genome gene expression profile by RNA-sequencing analysis. The potential signal pathway involved in the efficacy of 6-shogaol was also verified both in vivo and in vitro. We hope that this study will provide candidate targets for treating endotoxin-related hepatic disorders, as well as evidence for the therapeutic application of 6-shogaol for inflammatory liver diseases in the future.

## 2. Materials and Methods

### 2.1. Reagents

6-Shogaol with >98% purity was purchased from Nanjing Jingzhu Biotechnology Co. Ltd. (Nanjing, China). Dulbecco’s modified Eagle medium (DMEM), fetal bovine serum (FBS), penicillin, and streptomycin were purchased from Gibco Life Technologies (Grand Island, NY, USA). The nitrocellulose membrane was purchased from Bio-Rad Laboratories, Inc. (Hercules, CA, USA). BSA for blocking was purchased from VWR International, LLC. (Radnor, PA, USA). LPS from *Escherichia coli 055:B5* with a 99% purity was purchased from Solarbio Technology, Inc. (Beijing, China). Commercial kits for measuring levels of malondialdehyde (MDA), total superoxide dismutase (T-SOD), catalase (CAT), alanine aminotransferase (ALT), and aspartate aminotransferase (AST) were obtained from Nanjing Jiancheng Bioengineering Institute (Nanjing, China). Primary antibodies against β-actin, IκBα, phospho-IκBα(Ser32), p65, phospho-p65(Ser536), and secondary antibody horseradish peroxidase (HRP)-linked anti-rabbit antibody were purchased from Cell Signaling Technology (Danvers, MA, USA). Primary antibody against phospho-JNK1/JNK2/JNK3(Thr183/Thr183/Thr221) was purchased from Beyotime Biotechnology (Shanghai, China). Primary antibodies against JNK1, ERK1/2, phosphor-ERK1(Thr202/Tyr204)/ERK2(Thr185/Tyr187), and cellular protein extraction kit were obtained from Beijing Solarbio (Beijing, China). Pierce ECL Western Blotting Substrate was purchased from Thermo Fisher Scientific Inc. (Waltham, MA, USA). Monarch Total RNA Miniprep kit was purchased from New England BioLabs, Inc. (Ipswich, MA, USA). High-capacity cDNA reverse transcription kit was purchased from ThermoFisher Scientific (Waltham, MA, USA). The SYBR green reagents were purchased from Bio-Rad Laboratories, Inc. (Hercules, CA, USA).

### 2.2. Animals

A total of 24 eight-week-old ICR mice were purchased from Vital River Laboratory Animal Technology Co. Ltd. (Beijing, China). These mice were caged individually in a room maintained at 25 ± 1 °C with a relative humidity of 50 ± 10% and a light/dark cycle of 12 h. Water and food were free to access. The mice were divided randomly into three groups (*n* = 8): control, LPS, and 6-shogaol. 6-Shogaol was orally administered to mice in the 6-shogaol group at 20 mg/kg body weight for 7 consecutive days. The control and LPS groups were administered with the vehicle. On day 8, the mice in LPS and 6-shogaol groups were injected intraperitoneally with LPS (10 mg/kg body weight). After 24 h, the mice were sacrificed and the liver was dissected immediately. The procedure was authorized and approved by the institutional animal ethics committee (IQSTAP-RA-2019-09).

### 2.3. Biochemical Analysis

Serum was collected to measure ALT and AST activities using commercial kits. The livers were homogenized (10%, *w*/*v*) in cold normal saline. The prepared tissue homogenate was used for measuring SOD, and CAT activities, as well as MDA level using commercially available kits.

### 2.4. Histological Analysis

The liver tissues were fixed in 10% (*v*/*v*) formalin solution and embedded in paraffin wax. Then, the tissues were sliced (3 μm) and subjected to hematoxylin–eosin (HE) staining. All of the sections were observed under an Olympus BX60 microscope (Tokyo, Japan). The histological damage was scored in a blind manner based on lymphocytic cell infiltration (0–2), hydropic degeneration (0–2), and blood vessels congestion (0–2).

### 2.5. RNA-Sequencing Detection

Total RNA was isolated from liver tissues (3 samples per group) using TRIzol reagent (Invitrogen, Carlsbad, CA, USA). RNA quality was verified using Invitrogen Qubit 2.0 fluorometer. cDNA libraries preparation was subsequently performed with reagents supplied in Hieff NGS^TM^ MaxUp Dual-mode mRNA Library Prep Kit for Illumina according to the manufacturer’s instructions. The final purified libraries were quality evaluated, quantified, and sequenced using the BGI MGI 2000 platform according to the manufacturer’s instructions.

### 2.6. Bioinformatics Analysis

To obtain high-quality clean data for downstream analysis, the low-quality reads and reads containing adapters or ploy-N in raw data were removed. Differential expression of three groups was analyzed using R DESeq package 1.12.4. The threshold value of differentially expressed genes was set by |fold change| > 1.5 and *q*-value < 0.05.

Hierarchical cluster analysis was performed using R gplots package 2.17.0 to define differential gene expression patterns. GO and KEGG functional pathways were analyzed by R topGO and clusterProfiler packages for differentially expressed genes, respectively. Furthermore, the PPI network was screened based on the STRING database using the R igraph package.

### 2.7. Cell Culture and Treatment

BRL-3A cells, a fibroblast cell line derived from the liver of *Rattus norvegicus*, were obtained from the American Type Culture Collection (Manassas, VA, USA). Cells were cultured in DMEM complete medium containing 10% FBS at 37 °C in a thermostatic incubator with 5% CO_2_. When the cells were approximately 80% confluent, they were seeded in a 12-well plate and incubated for 24 h for adhesion. For quantitative real-time PCR (qPCR) analysis, the cells were pretreated with 6-shogaol for 12 h before being treated with LPS (0.1 μg/mL) for 5 h. For Western blot analysis, the cells were pretreated with 6-shogaol for 2 h before being treated with LPS (0.1 μg/mL) for 0.5 h. The dose for 6-shogaol was applied at 10 and 20 μM based on MTT assay and previous studies [13,19,20]. According to the MTT assay, 6-shogaol at 20 μM was found to be the highest concentration that did not show cytotoxicity to BRL-3A cells after treatment for 24 h (Supplementary Figure S1).

### 2.8. Quantitative Real-Time PCR Analysis for mRNA Expression

After treatments, total RNA from BRL-3A cells were extracted using a Monarch Total RNA Miniprep Kit (New England BioLabs Inc., Ipswich, MA, USA), and was reverse-transcripted using a high-capacity cDNA reverse transcription kit (ThermoFisher Scientific, Waltham, MA, USA). For the hepatic qPCR experiment, total RNA was isolated from the liver tissues using Trizol reagent, and reverse-transcribed using the high-capacity cDNA reverse transcription kit. The qPCR reactions were performed with an SYBR green PCR system in an ABI 7500 thermal cycler (Thermo Fisher Scientific, Waltham, MA, USA). Primers are synthesized by Sangon Biotech Co. Ltd. (Shanghai, China). The sequences are shown in Table 1. The cycling conditions were as follows: 95 °C for 1 min, followed by 40 cycles involving denaturing at 95 °C for 15 s and annealing at 60 °C for 60 s. Expression of mRNAs was normalized by the mRNA levels of β-actin, which was used as an internal control. The relative expression levels of mRNAs were analyzed using the 2^−ΔΔCt^ method.

### 2.9. Western Blot Analysis for Protein Expression

Proteins from BRL-3A cells were extracted using NP-40 lysis buffer with protease and phosphatase inhibitors added. Extraction of liver tissue protein fractions was performed as described by Das et al. [21]. Protein concentration was measured following the instructions of the BCA kit. An equal amount of protein (30 μg) was separated on 10% SDS–PAGE at a constant voltage of 100 V and transferred to nitrocellulose membranes under a current of 250 mA for 1.5 h. After blocking with 5% BSA, the membranes were incubated with primary antibodies overnight at 4 °C, followed by incubation with the HRP-linked secondary antibody at room temperature for 1 h. The blots were incubated with ECL substrate for 1 min. The signal intensities were visualized using the Tanon 5200 automatic chemiluminescence imaging system (Shanghai, China), and quantification of bands was performed using the Image J software (national institutes of health Bethesda, MD, USA).

### 2.10. Statistical Analysis

Results have been presented as means ± SD. Data differences were evaluated with a one-way analysis of variance (ANOVA), followed by Tukey’s test using Origin 8.0 (OriginLab, Northampton, MA, USA) differences were considered significant at *p* < 0.05.

## 3. Results

### 3.1. 6-Shogaol Improved Hepatic Function, Oxidative Stress, and Inflammation

**Figure 1:**
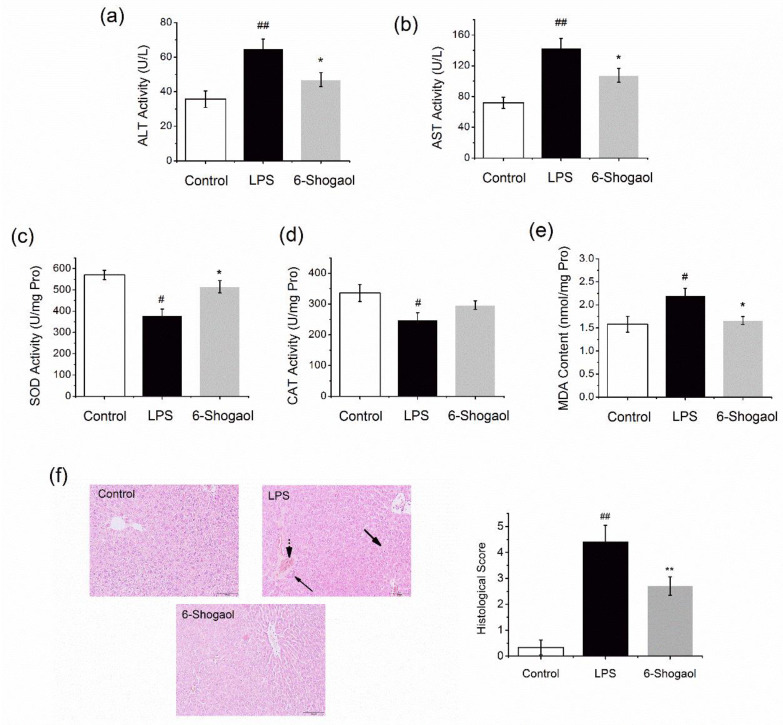
6-Shogaol improved hepatic function and oxidative stress in mice. ICR mice received daily oral administrations of 6-shogaol (20 mg/kg) for 7 consecutive days. The control and LPS groups were administered with the vehicle. On day 8, mice in LPS and 6-shogaol groups received an i.p. injection of LPS (10 mg/kg). After 24 h, the mice were sacrificed: (**a**) serum ALT activity; (**b**) serum AST activity; (**c**) hepatic SOD activity; (**d**) hepatic CAT activity; (**e**) hepatic MDA level; (**f**) histological examination of liver tissues by HE staining. LPS group showed lymphocytic infiltration (arrow), hydropic degeneration (thick arrow), and congested blood vessels (arrow with dot tail). Data are presented as mean ± SEM (*n* = 8). # *p* < 0.05, ## *p* < 0.01 versus control group; * *p* < 0.05 versus LPS group, ** *p* < 0.01 versus LPS group. LPS, lipopolysaccharide; ALT, alanine aminotransferase; AST, aspartate aminotransferase; SOD, superoxide dismutase; CAT, catalase; MDA, malondialdehyde.

In order to examine the hepatoprotective effect of 6-shogaol in vivo, animal experiments were performed. We observed that LPS induced liver injury in mice characterized by elevated ALT and AST levels (Figure 1a,b, *p* < 0.05). 6-Shogaol significantly protected the liver function by reducing these levels (*p* < 0.05). As shown in Figure 1c–e, LPS treatment evoked oxidative stress in the liver, presented by decreased SOD and CAT activities, and elevated MDA levels (*p* < 0.05). Treatment with 6-shogaol enhanced the cellular antioxidant defense system by elevating SOD and CAT activities (*p* < 0.05 for SOD). Moreover, hepatic MDA level was markedly attenuated by 6-shogaol treatment (Figure 1e, *p* < 0.05). LPS-induced liver injury was confirmed by the morphological alterations of the liver by HE staining. As shown in Figure 1f, liver tissue sections from LPS-exposed mice displayed lymphocytic infiltration, cellular hydropic degeneration, and congested blood vessels. Obvious reductions in these pathological alterations were observed in the 6-shogaol-treated group, i.e., hepatic sections were nearly normal except for moderate hydropic degeneration. The histological score for the 6-shogaol group decreased 38.6%, compared with LPS alone (*p* < 0.01). These data suggest that 6-shogaol treatment may improve LPS-induced liver function, oxidative stress, and inflammation.

### 3.2. 6-Shogaol Suppressed MAPK/NFκB Pathway in the Liver

**Figure 2:**
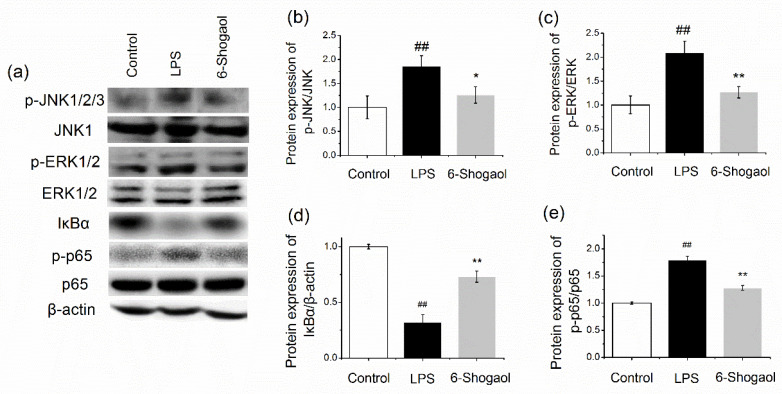
6-Shogaol inhibited hepatic inflammation through MAPK/NFκB pathway. ICR mice received daily oral administrations of 6-shogaol (20 mg/kg) for 7 consecutive days. The control and LPS groups were administered with the vehicle. On day 8, mice in LPS and 6-shogaol groups received an i.p. injection of LPS (10 mg/kg). After 24 h, the mice were sacrificed: (**a**) the blots; (**b**) hepatic protein expression of p-JNK/JNK; (**c**) hepatic protein expression of p-ERK/ERK; (**d**) hepatic protein expression of IκBα; (**e**) hepatic protein expression of p-p65/p65. Data are presented as mean ± SEM (*n* = 3). ## *p* < 0.01 versus control group; * *p* < 0.05, ** *p* < 0.01 versus LPS group.

Activation of MAPK/NFκB is crucial for inflammatory response. Therefore, we next evaluated the protein expression of JNK, ERK, IkBα, and NFκB p65 (Figure 2). LPS activated MAPK by significantly elevating the phosphorylation of JNK and ERK, thereby caused degradation of IκBα and triggered the phosphorylation of p65 (*p* < 0.01). All these changes were markedly reversed by treatment with 6-shogaol (*p* < 0.05 and *p* < 0.01). These results indicate that 6-shogaol regulated hepatic inflammation through MAPK/NFκB pathway.

### 3.3. Transcriptional Response in the Liver after 6-Shogaol Treatment

**Figure 3:**
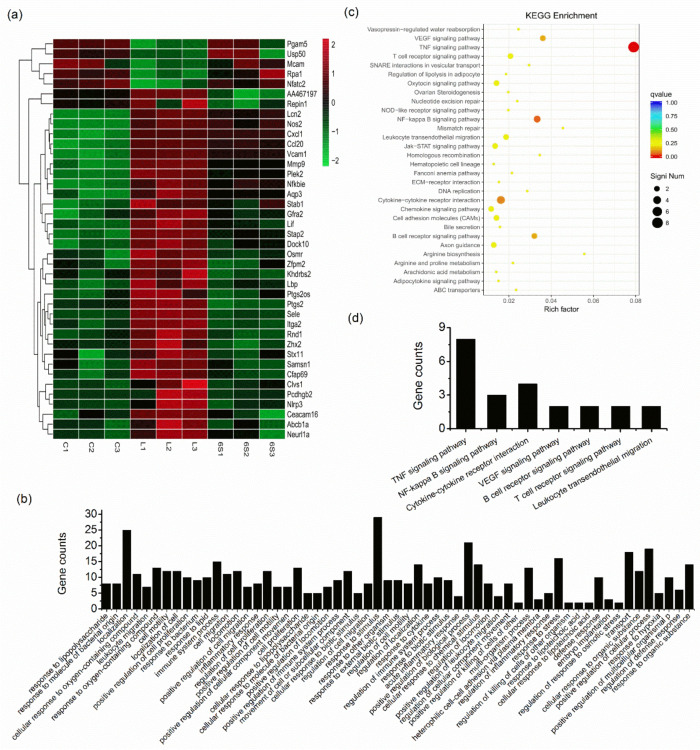
Transcriptomic analysis for mice liver tissues. ICR mice received daily oral administrations of 6-shogaol (20 mg/kg) for 7 consecutive days. The control and LPS groups were administered with the vehicle. On day 8, mice in LPS and 6-shogaol groups received an i.p. injection of LPS (10 mg/kg). After 24 h, the mice were sacrificed: (**a**) hierarchical cluster heatmap for the overlap differentially expressed genes based on expression values (log_2_(TPM + 1)). Samples are displayed in columns and genes in rows. The color represents gene expression level, i.e., brighter red indicates higher expression values and brighter green indicates lower expression values; (**b**) GO (biological process) terms enriched for overlapped differentially expressed genes; (**c**) KEGG pathway enrichment for overlapped differentially expressed genes. The top 30 pathways are presented. The Y-axis and X-axis indicate functional pathways and the enrich factor for the differentially expressed genes, respectively; (**d**) gene counts in each enriched pathway (presenting pathways with gene counts >2).

To further investigate the target genes regulated by 6-shogaol, RNA sequencing was performed. As shown in Figure 3, LPS- and LPS/6-shogaol-treated mice showed differential gene profiles in the liver. Using the cutoff false discovery rate <0.05 and fold change >1.5 (upregulated) or <0.67(downregulated), we identified 41 overlapped genes between the LPS vs. control group and 6-shogaol vs. LPS group, among which 36 genes were upregulated, and 5 genes were downregulated for LPS vs. control group (Table 2). We performed hierarchical cluster analysis for the overlapped genes and observed distinctly different expression patterns (Figure 3a). All of these genes are differentially expressed in the opposite patterns in 6-shogaol vs. LPS group, e.g., Ptgs2, Mmp9, Ccl20 are upregulated by LPS, whereas downregulated in the 6-shogaol group.

### 3.4. Gene Functional Analysis

**Figure 4:**
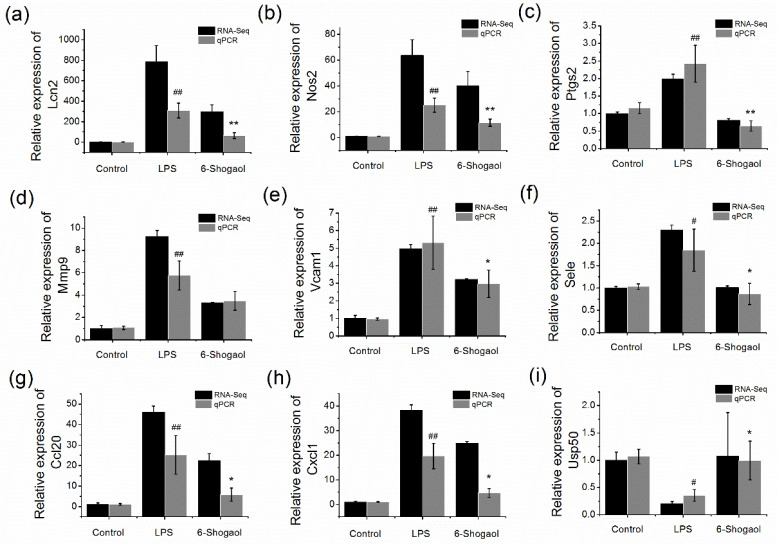
Validation of transcriptome data by qPCR: (**a**) mRNA expression of Lcn2; (**b**) mRNA expression of Nos2; (**c**) mRNA expression of Ptgs2; (**d**) mRNA expression of Mmp9; (**e**) mRNA expression of Vcam1; (**f**) mRNA expression of Sele; (**g**) mRNA expression of Ccl20; (**h**) mRNA expression of Cxcl1; (**i**) mRNA expression of Usp50. Data are presented as mean ± SEM (*n* = 3). # *p* < 0.05, ## *p* < 0.01 versus control group; * *p* < 0.05, ** *p* < 0.01 versus LPS group.

Functional analysis was performed for the overlapped differentially expressed genes to examine whether they have biological relevance. These genes were classified into gene ontology (GO) terms such as positive regulation of immune system process, cellular response to lipopolysaccharide, and positive regulation of biological process (Figure 3b). Pathway enrichment analysis revealed that 21 overlapped genes were enriched in Kyoto Encyclopedia of Genes and Genomes (KEGG) signaling pathways including the TNF signaling pathway and NFκB signaling pathway (Figure 3c,d). In particular, Ccl20, Cxcl1, Mmp9, Vcam1, Sele, Lif, Ptgs2, and Pgam5 were enriched in the TNF signaling pathway; Vcam1, Ptgs2, Lbp were enriched in NFκB signaling pathway. Except for Pgam5, all the above genes were upregulated in the LPS group, which were significantly decreased by 6-shogaol. The detailed information for the pathway enrichment was listed in Table 3. Expression of representative differentially expressed genes was evaluated by qPCR analysis. The results show similar expression patterns, as observed with RNA sequencing, which validated our transcriptome data (Figure 4). These results revealed that the overlapped differentially expressed genes were mainly focused on inflammation-related functions.

### 3.5. Protein–Protein Interaction (PPI) Network for the Overlapped Genes

**Figure 5:**
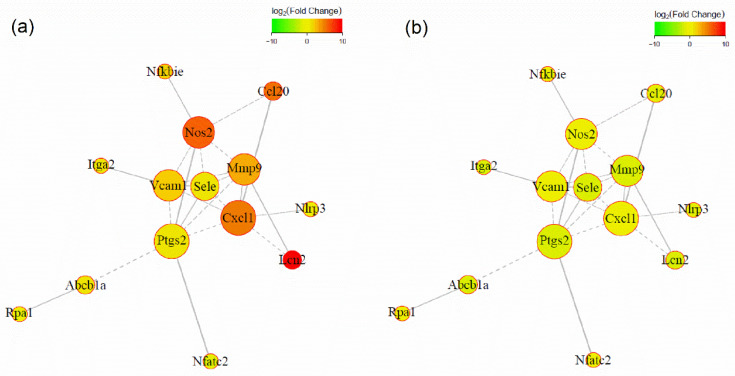
PPI network for the overlapped differentially expressed genes: (**a**) LPS vs. control group; (**b**) 6-shogaol vs. LPS group. Nodes represent proteins and edges represent interactions between two proteins. The size of proteins indicates node degree value. Solid lines indicate that the relationship between genes has been verified, and dashed lines indicate that it has not.

PPI network of the overlapped differentially expressed genes is shown in Figure 5. In the network, nodes represent proteins and edges represent interactions between two proteins. The size of proteins indicates node degree value, i.e., the larger the node is, the more important it is in the network. The color of the nodes indicates fold change for different comparison groups. We observed 14 differentially expressed genes encoding proteins were involved in the network, among which, NOS2, VCAM1, E-Selectin, MMP9, PTGS2, CXCL1 are important hub proteins. These proteins were linked primarily to inflammation and cell adhesion.

### 3.6. 6-Shogaol Suppressed the mRNA Expression of Overlapped Genes in LPS-Induced BRL-3A Cells

**Figure 6:**
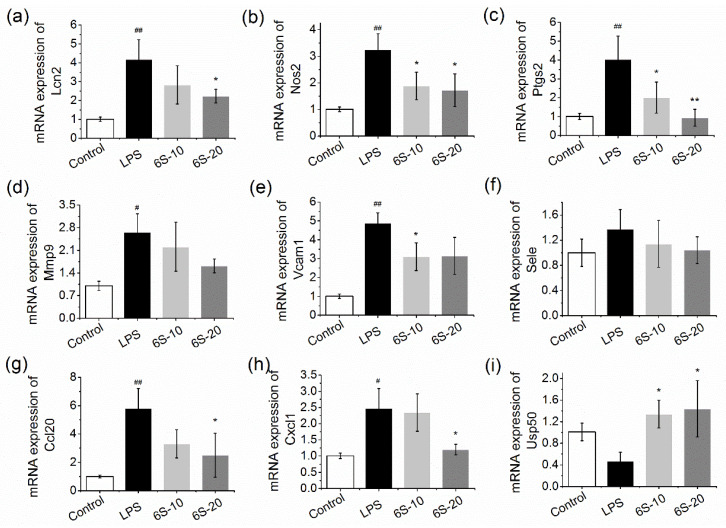
6-Shogaol inhibited overlapped gene mRNA expression in LPS-treated BRL-3A cells. BRL-3A cells were pre-treated with 6-shogaol for 12 h before treated with LPS (0.1 μg/mL) for 5 h: (**a**) mRNA expression of Lcn2; (**b**) mRNA expression of Nos2; (**c**) mRNA expression of Ptgs2; (**d**) mRNA expression of Mmp9; (**e**) mRNA expression of Vcam1; (**f**) mRNA expression of Sele; (**g**) mRNA expression of Ccl20; (**h**) mRNA expression of Cxcl1; (**i**) mRNA expression of Usp50. 6S-10, 10 μM of 6-shogaol; 6S-20, 20 μM of 6-shogaol. Data are presented as mean ± SEM (*n* = 6). # *p* < 0.05, ## *p* < 0.01 versus control group; * *p* < 0.05, ** *p* < 0.01 versus LPS group.

To evaluate whether 6-shogaol has the same effect in vitro, the mRNA expression of the regulated genes was also analyzed in LPS-induced BRL-3A cells. The results show that the differentially expressed genes exhibited similar expression patterns in LPS-treated BRL-3A cells (Figure 6). 6-Shogaol dose dependently and effectively reversed the upregulation/downregulation by LPS. 6-Shogaol at high dose markedly decreased mRNA expression of Ptgs2 to the level even slightly lower than the control group (*p* < 0.01). Moreover, it significantly elevated Usp50 mRNA expression by over twofold, compared to the LPS-treated group (*p* < 0.01). For mRNA expression of Lcn2, Nos2, Ccl20, and Cxcl1, 6-shogaol at a high dose could induce approximately 50% reduction, compared to the LPS-treated group (*p* < 0.05).

### 3.7. 6-Shogaol Inhibited NFκB and MAPK Pathway in BRL-3A Cells

**Figure 7:**
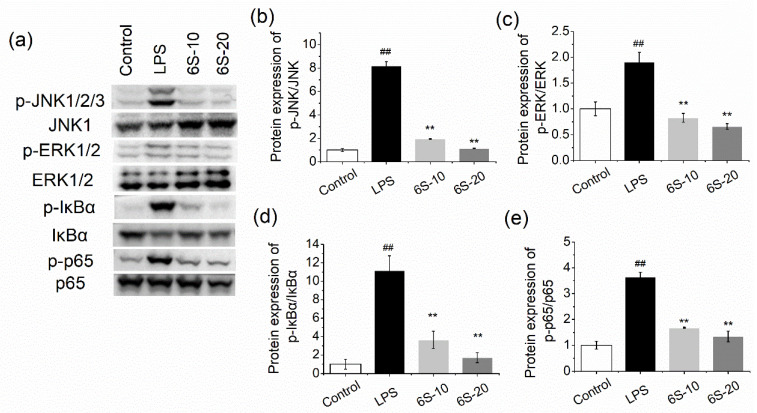
6-Shogaol inhibited MAPK/NFκB pathway in LPS-treated BRL-3A cells. The cells were pretreated with 6-shogaol for 2 h before being treated with LPS (0.1 μg/mL) for 0.5 h: (**a**) the blots; (**b**) protein expression of p-JNK/JNK; (**c**) protein expression of p-ERK/ERK; (**d**) protein expression of p-IκBα/IκBα; (**e**) protein expression of p-p65/p65. 6S-10, 10 μM of 6-shogaol; 6S-20, 20 μM of 6-shogaol. Data are presented as mean ± SEM (*n* = 3). ## *p* < 0.01 versus control group; ** *p* < 0.01 versus LPS group.

We next investigated the effect of 6-shogaol on protein expressions of MAPK and NFκB pathways in BRL-3A cells. As shown in Figure 7a–c, LPS significantly activated the MAPK pathway, with an 8.2-fold and 1.9-fold increase in p-JNK/JNK and p-ERK/ERK expressions, respectively, compared to the control cells. Moreover, phosphorylation of IκBα was markedly elevated in LPS-treated cells (*p* < 0.01), which promoted degradation of IκBα and activation of NFκB (Figure 7a,d,e). 6-Shogaol evidently blocked the activation of MAPK and NFκB pathways, the expression levels of p-JNK/JNK, p-ERK/ERK, p-IκBα/IκBα, and p-p65/p65 were reduced to the level comparable to or even lower than the control cells (*p* < 0.01). These results are in accordance with those in the in vivo study.

## 4. Discussion

A murine model of LPS-induced inflammatory liver injury aims to replicate sepsis-induced acute hepatic injury in humans. It is widely used to investigate potential therapeutics for the treatment of acute hepatic failure [22,23,24]. In the present study, administration of LPS results in an impaired structure of the liver and obvious histological changes including lymphocytic infiltration, cellular hydropic degeneration, and congested blood vessels. These histological changes were markedly ameliorated by 6-shogaol treatment. In addition, 6-shogaol evidently reduced serum ALT and AST activities. These results demonstrated the potency of 6-shogaol in treating inflammatory liver injuries. The present study further investigated the possible protection mechanism offered by oral administration of 6-shogaol, which is not yet reported by other reports.

The impairment of the antioxidant defense system is a critical step in LPS-induced injury. Evidence has shown that LPS insult is characterized by a change in the level of tissue antioxidant enzyme and increment of ROS production in the liver [25]. Restoring the antioxidant system may be a good choice to reverse liver injury [26]. As a main O^2−^ scavenger, SOD may react with ROS and NO, and produce H_2_O_2_, which is degraded into oxygen and water by CAT. In the current study, we found a modulation of the antioxidant system in the LPS-induced mice consuming 6-shogaol, with the normalization of SOD and CAT activities. The enhanced antioxidant system may help prevent LPS-induced liver damage.

LPS-induced liver injury was highly associated with inflammatory response [27]. A previous study has proposed that 6-shogaol is beneficial in suppressing inflammatory response in LPS-induced macrophages [17]. Our present study demonstrated that 6-shogaol possesses a similar effect in LPS-challenged hepatocytes. NFκB signaling is a central regulator of an inflammatory response, which regulates inflammatory cytokines and mediator genes expression [28]. In resting cells, the NFκB is binding with its inhibitory proteins IκBα. With exposure to proinflammatory stimuli, IκBα becomes phosphorylated and degraded. Then, the liberated NFκB is translocated to the nuclei and induces the transcription of target genes. In the present study, LPS induced the degradation of IκBα and significantly elevated phosphorylated p65 protein expression (Figure 2 and Figure 6), which indicated that the NFκB pathway is activated. 6-Shogaol significantly inhibits hepatic inflammatory injury by suppressing the activation of the NFκB pathway. MAPK pathway has been proposed to respond to cytokines and stress, which are always related to the inflammation signaling pathway [29]. Particularly, MAPKs play an important role in modulating the NFκB pathway [30]. Therefore, protein expressions of MAPK are detected. LPS induces activation of MAPKs via phosphorylating JNK and ERK. Additionally, we confirmed the inhibitory effect of 6-shogaol on MAPKs. These results suggest that the protective effect of 6-shogaol on LPS-injured hepatocytes is related to the suppression of the MAPK/NFκB pathway.

We further investigated targeted genes involved in 6-shogaol treatment by applying RNA-sequencing analysis. We observed that 6-shogaol alleviates LPS-induced hepatic inflammation by altering a batch of gene expression, most of which were involved in the TNF and NFκB signaling pathways by KEGG pathway analysis. This is consistent with our results that 6-shogaol inhibits the NFκB pathway both in vivo and in vitro by Western blot analysis (Figure 2 and Figure 7).

Lipocalin-2 (LCN-2) in the liver shows a protective role against pernicious conditions, such as infection, inflammation, and other forms of cellular stress [31]. There is a rapid and sustained LCN2 production in injured hepatocytes, which is regulated by the NFκB pathway [32,33]. As expected, LPS-induced a 785-fold increase in the expression level of Lcn2 in the liver of mice by RNA sequencing. This elevation was markedly reversed by 6-shogaol treatment. These results suggest that LCN2 is a potential biomarker for hepatic inflammation, as well as a viable clinical target for treating sepsis-related hepatic injuries.

NO is a critical factor in the pathogenesis of inflammatory diseases. The majority of NO production under the inflammatory state derives from inducible nitric oxide synthase (NOS2) [34]. Ptgs2 encodes the protein expression of cyclooxygenase-2 (COX-2), which is responsible for the synthesis of prostaglandin E2. Elevation in NOS2 and Ptgs2 expression by LPS via the NFκB pathway is well documented in various in vivo and in vitro models [35,36,37]. We proved that NOS2 and Ptgs2 play critical roles in hepatocyte inflammation evoked by LPS. These elevations could be effectively attenuated by treatment of 6-shogaol, which is consistent with other studies [17,38].

Adhesion molecules, such as vascular cell adhesion molecule 1 (VCAM-1) and E-selectin, have been shown to involve in the inflammation response promoted by TNF-α via activating the NFκB signaling pathway [39,40]. Chemokines are also downstream targets of the NFκB pathway [41,42]. They play an important role in the pathogenesis of hepatic diseases. Ccl20 and Cxcl1 mediate LPS-induced liver injury and are potential drivers of inflammation and fibrosis in alcoholic hepatitis [43,44,45]. All of the aforementioned genes were downregulated by 6-shogaol, which is rarely reported by other researchers.

Matrix metalloproteinases (MMPs) are responsible for the degradation and remodeling of the extracellular matrix proteins. They participate in many pathological processes, such as inflammation and tumor invasion. Mmp9 is a downstream effector of the NFκB pathway [46]. Elevation in Mmp9 expression was frequently observed in reported LPS-induced cell models [47,48]. However, Zhang et al. demonstrated that overexpression of Mmp9 plays a protective role against LPS-induced inflammation in osteoblasts [49]. In the present study, we observed that Mmp9 was upregulated in LPS-treated mice/cells and remarkably reduced by 6-shogaol treatment. Therefore, future investigations are warranted to safely include Mmp9 into viable clinical treatments for sepsis-related inflammation.

## 5. Conclusions

Our findings demonstrated, for the first time, that 6-shogaol shows the preventive effect in sepsis-associated acute liver injury via enhancing antioxidant defense system and suppressing MAPK/NFκB induced inflammatory responses. Transcriptomic analysis revealed that the efficacy of 6-shogaol involves the regulation on Lcn2, Nos2, Ccl20, Cxcl1, Mmp9, Vcam1, Sele, Ptgs2, and Usp50 mRNA expression. 6-Shogaol shows great potential as a natural chemopreventive agent and may be used in the future to treat sepsis-associated hepatic disorders

## Figures and Tables

**Table 1:** Primer information of qPCR amplification.

Genes	Primer Sequences
*Mus musculus*	
*Lcn2*	F: GGGCTGTCGCTACTGGAT
	R: TTGGTATGGTGGCTGGTG
*Nos2*	F: ACATGCAGAATGAGTACCGG
	R: TCAACATCTCCTGGTGGAAC
*Ccl20*	F: ACTGTTGCCTCTCGTACATACA R: GAGGAGGTTCACAGCCCTTTT
*Cxcl1*	F: TGGCTGGGATTCACCTCAAGA R: TGTGGCTATGACTTCGGTTTGG
*Mmp9*	F: AAGGGTACAGCCTGTTCCTGGT R: CTGGATGCCGTCTATGTCGTCT
*Vcam1*	F: CTGGGAAGCTGGAACGAAGT R: GCCAAACACTTGACCGTGAC
*Sele*	F: TCAACTTGAGTGCACATCTCAGG
	R: TGATTGAAGGCT TTGGCAGCT
*Ptgs2*	F: CAATACTGGAAGCCGAGCAC
	R: CAGCTCAGTTGAACGCCTTT
*β-actin*	F: GTGACGTTGACATCCGTAAAGA
	R: GCCGGACTCATCGTACTCC
*Usp50*	F: GCCTACTACAACCTTGCGGAG R: TCCACCAGTGGAGATACGCT
*Rattus norvegicus*	
*Lcn2*	F: GATTCGTCAGCTTTGCCAAGT
	R: CATTGGTCGGTGGGAACAG
*Nos2*	F: GATCAATAACCTGAAGCCCG
	R: GCCCTTTTTTGCTCCATAGG
*Ccl20*	F: CACTGAGCAGATCAATTCCTGGAG R: TGTACGTGAGGCAGCAGTCAAAG
*Cxcl1*	F: AAATGGTGAAGGTCGGTGTGAAC R: CAACAATCTCCACTTTGCCACTG
*Mmp9*	F: TCGAAGGCG ACCTCAAGTG
	R: TTCGGTGTAGCTT TGGATCCA
*Vcam1*	F: GTATACGAGTGTGAATCGAAAACCG R: CAAGGAGTTCAGGGGAAAAATAGTC
*Sele*	F: TCAACTTGAGTGCACATCTCAGG
	R: TGATTGAAGGCT TTGGCAGCT
*Ptgs2*	F: TTCGGGAGCACAACAGAGTG
	R: TGAAGTGGTAACCGCTCAGG
*Usp50*	F: GTGCCTACTACATCCTTGCG R: CATGTAGCAGGTGTTGCCCA
*β-actin*	F: GTGACGTTGACATCCGTAAAGA
	R: GCCGGACTCATCGTACTCC

**Table 2:** The overlapped differentially expressed genes list.

Gene Name	LPS vs. Control		6-Shogaol vs. LPS	
	FoldChange	*q* Value	FoldChange	*q* Value
Lcn2	785.327	0.000	0.382	0.000
Pcdhgb2	88.845	0.000	0.016	0.000
Nos2	63.652	0.000	0.630	0.019
Ccl20	45.973	0.000	0.489	0.000
Cxcl1	38.367	0.000	0.652	0.000
Mmp9	9.272	0.000	0.356	0.000
Stab1	7.088	0.000	0.477	0.000
Vcam1	4.968	0.000	0.649	0.000
Nfkbie	3.755	0.000	0.659	0.004
Samsn1	3.450	0.045	0.287	0.002
Aqp3	2.911	0.000	0.602	0.001
Stap2	2.782	0.000	0.564	0.000
Cfap69	2.532	0.000	0.420	0.014
Abcb1a	2.412	0.001	0.434	0.003
Sele	2.304	0.000	0.439	0.000
Dock10	2.248	0.000	0.603	0.005
Plek2	2.219	0.000	0.659	0.000
Itga2	2.153	0.000	0.441	0.000
Lif	2.055	0.000	0.588	0.000
Gfra2	2.041	0.000	0.616	0.002
Ptgs2	1.984	0.000	0.406	0.000
Khdrbs2	1.861	0.038	0.449	0.001
Clvs1	1.801	0.013	0.611	0.041
Nlrp3	1.759	0.000	0.583	0.001
Lbp	1.707	0.001	0.655	0.004
Neurl1a	1.686	0.007	0.620	0.009
Osmr	1.644	0.000	0.649	0.000
AA467197	1.640	0.016	0.322	0.000
Ceacam16	1.634	0.009	0.569	0.005
Ptgs2os	1.616	0.011	0.645	0.022
Rnd1	1.590	0.000	0.604	0.000
Stx11	1.584	0.000	0.657	0.000
Zhx2	1.535	0.001	0.571	0.000
Zfpm2	1.533	0.020	0.656	0.014
Repin1	1.514	0.013	0.384	0.001
Dock10	2.248	0.000	0.603	0.005
Rpa1	0.618	0.029	1.628	0.000
Pgam5	0.594	0.000	1.505	0.008
Mcam	0.583	0.043	1.595	0.041
Nfatc2	0.539	0.001	1.703	0.000
Usp50	0.205	0.000	5.262	0.027

**Table 3:** The overlapped differentially expressed genes pathway enrichment.

KEGG Pathway Name	Genes Enriched in Pathway	*p* Value
TNF signaling pathway	Ccl20, Cxcl1, Mmp9, Vcam1, Sele, Lif, Ptgs2, Pgam5	0.000
NFκB signaling pathway	Vcam1, Ptgs2, Lbp	0.002
Cytokine–cytokine receptor interaction	Ccl20, Cxcl1, Lif, Osmr	0.006
VEGF signaling pathway	Ptgs2, Nfatc2	0.012
B cell receptor signaling pathway	Nfkbie, Nfatc2	0.015
T cell receptor signaling pathway	Nfkbie, Nfatc2	0.035
Leukocyte transendothelial migration	Mmp9, Vcam1	0.042

## Supplementary Materials

The following are available online at https://www.mdpi.com/article/10.3390/nu13103427/s1, Figure S1: Effect of 6-shogaol on cell viability. BRL-3A cells were treated with different concentrations of 6-shogaol for 24 h, and cell viability was measured using an MTT assay.
