# Supplementary material for: 6-Shogaol Mitigates Sepsis-Associated Hepatic Injury through Transcriptional Regulation
Source: Nutrients. 2021 Sep 28;13(10):3427. doi: 10.3390/nu13103427 (PMC8540559; doi:10.3390/nu13103427)
Supplement: Supplementary file 1 [file nutrients-13-03427-s001.zip › nutrients-1358507-supplementary.pdf]

Materials and Methods

Reagents

MTT was obtained from Sigma-Aldrich, Inc. (St Louis, MO, USA).

MTT assay

Briefly, BRL-3A cells were seeded at a density of 5×10^4 cells/ml in 96-well plates. After 24h, cells were treated with 6-shogoal (0, 5, 10, 20, 30, 40, 50 μM). The plates were then incubated at 37℃ for 24 h. 20 μL of MTT solution (5 mg/mL) was added to each well and the cells were cultured for another 2 h. Then the medium was discarded, and the formazan crystals were dissolved with DMSO. The optical density was read at 490 nm using a microplate reader (TECAN, Männedorf, Switzerland). Cell proliferation rates were calculated by comparing with the control cells.

Results

Effect of 6-shogoal on cell viability

As shown in Figure 1S, 5-20 μM of 6-shogaol were not cytotoxic to the BRL-3A cells after 24 h of treatment. However, the cytotoxicity of 6-shogaol was observed at a concentration higher than 30 μM ($p < 0.05$). Thus, we used 10 and 20 μM of 6-shogoal in the following in vitro study.

Figure Legend

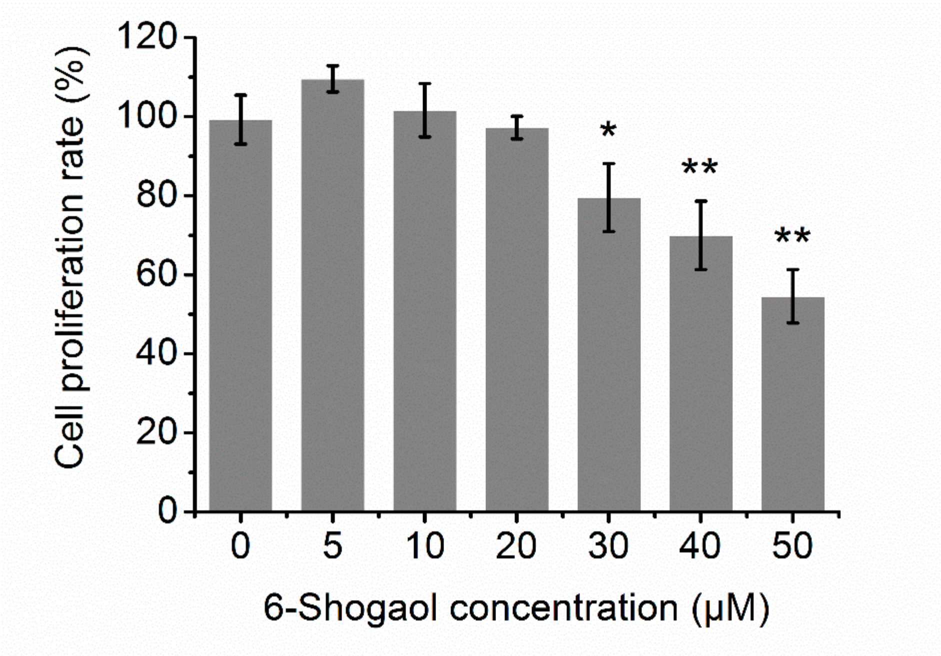

Figure S1. Effect of 6-shogaol on cell viability. BRL-3A cells were treated with different concentrations of 6-shogoal for 24 h, and cell viability was measured using MTT assay. Data are presented as mean ± SD (n = 6). **p* < 0.05 vs. cells in 0 μM group; ***p* < 0.01 vs. cells in 0 μM group.
